# Non-linear association and benchmark dose of blood pressure on carotid artery intima-media thickening in a general population of southern China

**DOI:** 10.3389/fcvm.2024.1325947

**Published:** 2024-05-13

**Authors:** Linyuan Qin, Xiaoyan Wu, Chao Tan, Zhengbao Zhang, You Li, Xiaonian Zhu, Shenghua Qin, Shengkui Tan

**Affiliations:** ^1^ Department of Epidemiology and Health Statistics, School of Public Health, Guilin Medical University, Guilin, Guangxi, China; ^2^ Guangxi Key Laboratory of Environmental Exposomics and Entire Lifecycle Health, Guilin Medical University, Guilin, Guangxi, China; ^3^ Physical Examination Center, Guilin People's Hospital, Guilin, Guangxi, China; ^4^ Party Committee Office, Youjiang Medical University for Nationalities, Baise, Guangxi, China

**Keywords:** carotid artery intima-media thickening, blood pressure, restricted cubic splines, benchmark dose, non-linear association

## Abstract

**Background and aims:**

This study aimed to evaluate whether there is a J-curve association between blood pressure (BP) and carotid artery intima-media thickening (CAIT) and estimate the effect of the turning point of BP on CAIT.

**Methods and results:**

Data from 111,494 regular physical examinations conducted on workers and retirees (aged 18 years or older) between January 2011 and December 2016, exported from the hospital information system, were analyzed. Restricted cubic splines (RCS) logistic regression was employed to access the association of BP with CAIT, and Bayesian benchmark dose methods were used to estimate the benchmark dose as the departure point of BP measurements. All the *p*_non-linear_ values of BP measurements were less than 0.05 in the RCS logistic regression models. Both systolic blood pressure (SBP) and diastolic blood pressure (DBP) had J-curve associations with the risk of CAIT at a turning point around 120/70 mmHg in the RCS. The benchmark dose for a 1% change in CAIT risk was estimated to be 120.64 mmHg for SBP and 72.46 mmHg for DBP.

**Conclusion:**

The J-curve associations between SBP and DBP and the risk of CAIT were observed in the general population in southern China, and the turning point of blood pressure for significantly reducing the risk of CAIT was estimated to be 120.64/72.46 mmHg for SBP/DBP.

## 1 Introduction

Since their report in 2005, cardiovascular disease and stroke have been the major causes of mortality in China (1). Blood pressure (BP), which is the leading modifiable risk factor, plays a very important role in the development of these cardiovascular diseases and is also associated with carotid artery intima-media thickening (CAIT) (2). Previous studies have provided evidence that interventions aimed at achieving persistent BP reduction in individuals with elevated BP could considerably benefit primary prevention efforts against cardiovascular disease and stroke (3, 4). Early before the onset of cardiovascular disease and stroke, vessel damage caused by elevated BP could be screened through CAIT (5, 6), which could be detected by carotid ultrasound examination during routine physical examinations of the general population. Therefore, exploring the relationship between BP and CAIT can provide a basis for earlier prevention of cardiovascular diseases and stroke. Even though previous studies had shown that high BP is an independent risk factor for CAIT (7–12), most of them usually accessed the generalized linearity associations between the risk of CAIT and BP. However, in real-world data, non-linear associations warrant consideration because a unit (e.g., 20 mmHg) increases in BP at a higher BP level, and the change in the risk of CAIT is not the same as it is at a lower BP level (2). Furthermore, from a micro perspective, platelet microparticle values, which are positively associated with CAIT (13, 14), are elevated in the higher BP hypertension group but not in the mild BP hypertension and normotension groups (15). The J-curve association between the risk of cardiovascular disease or stroke and BP has been observed for decades (16–18). These research studies suggested potential non-linear associations between BP and CAIT, yet the details of the picture remain unclear.

At present, the antihypertensive targets recommended in hypertension guidelines are based on the principle of significantly reducing the risk of cardiovascular and cerebrovascular diseases (19). As one of the major arteries closest to the heart, the presence of CAIT indicates that atherosclerosis may also affect cardiovascular or cerebral vessels (20, 21). Therefore, evaluating a BP turning point that can significantly reduce the risk of CAIT as a BP target would be more beneficial for managing BP in the general population. This paper aimed to explore whether there are J-curve associations between BP measurements and CAIT and to estimate the turning points of systolic blood pressure (SBP) and diastolic blood pressure (DBP) through longitudinal data analysis, thus improving hypertension management and preventing cardiovascular disease and stroke.

## 2 Materials and methods

### 2.1 Study population and data collection

Data from regular physical examinations conducted on workers and retirees (18 years of age or older) between January 2011 and December 2016 were exported from the hospital information system at the Affiliated Hospital of Guilin Medical University in Southern China. These data, which covered 111,494 examinations of 32,276 people who had participated at least twice in annual examinations and did not have CAIT at the first examination, included 41 variables that comprised routine blood tests (including complete blood count and chemistry panels for basic metabolic, liver function, and kidney function), BP readings, and ultrasound radiography results. Hypertension patients regularly taking antihypertensive medications (including beta-blockers, diuretics, angiotensin-converting enzyme inhibitors, and angiotensin II receptor blockers) were excluded from the study.

CAIT was assessed by high-resolution B-mode ultrasonography of the right and left carotid arteries of a 1–1.5 cm section at the distal end of the proximal to the carotid bulb. A mean intima-media thickness >1.0 mm was diagnosed as CAIT. Four deputy chief physicians in the department of ultrasound were responsible for the diagnosis of CAIT using an ultrasound imager (G4 xMATRIX iU22, Phillips). BP levels were measured with an automatic cuff-style bicep monitor (HEM-1000, Omron) after the participants rested for at least 5 min. The average of two readings on two occasions was used as the BP level. SBP and DBP were used to compute the following BP measurements: pulse pressure (PP: SBP − DBP) and mean arterial pressure (MAP: 2/3 × DBP + 1/3 × SBP). To facilitate better BMD model fit and comparison with other studies, BP measurements were transformed into groups. SBP was categorized into groups of <100, ∼100, ∼120, ∼140, ∼160, and ≥180 mmHg, while DBP was categorized into groups of <60, ∼60, ∼70, ∼80, ∼90, ∼100, and ≥110 mmHg as in the INVEST study (22). PP was grouped as <35, ∼35, ∼45, ∼55, ∼65, and ≥75 mmHg, while MAP was grouped as <70, ∼70, ∼80, ∼90, ∼100, ∼110, and ≥120 mmHg.

### 2.2 Covariate variables

The potential covariate variables in this study were identified based on subjective knowledge, literature reviews (23–28), and three elastic net models (see Supplementary Materials). Covariates included age, sex, BMI, fasting blood glucose, low-density lipoprotein cholesterol, serum uric acid, albumin, alkaline phosphatase, homocysteine, and fatty liver, which were used as adjustment variables.

### 2.3 Statistical analysis

The extreme outlier values (more extreme than Q1 − 3 × IQR or Q3 + 3 × IQR) were treated as missing data. The missing continuous values for an individual were imputed using the mean values of their longitudinal data if they could be calculated; otherwise, the mean values of data from participants of the same age and sex were used as substitutes. The observations with missing CAIT data were excluded. Continuous variables were presented as mean with standard deviation (for normally distributed data) or median (P25–P75) (for skewed distributed data), while categorical variables were described as number (*n*) and percentage (%). Comparisons of continuous variables between groups were done using *t*-tests or Mann–Whitney *U*-tests according to their skewness. To avoid multicollinearity, we used SBP, DBP, PP, and MAP separately as the main explanatory variables, with the CAIT condition (yes vs. no) serving as the dependent variable to fit the models. To access the non-linear association between BP and the risk of CAIT, we added restricted cubic splines (RCS) terms (set to three knots) of BP measurements to logistic models, with all the covariate variables adjusted. The results of the RCS logistic models were presented as spline curves based on main effects with 95% confidence intervals (CIs). Benchmark dose (BMD) models without adjusting for the covariate variables were used to assess the turning point for the risk of BP measurements on CAIT. To balance the weights of each model, we employed the Bayesian benchmark dose model (BBMD^1^) (29) to evaluate the BMD and the lower limit of BMD (BMDL) at benchmark response (BMR) of 1%, 5%, 10%, and 20% prevalence change of CAIT and the risk at different levels of BP measurements. The midpoint values of groups of BP measurements were used to estimate the turning point in BBMD. Values at the lower end of each BP measurement were set to the lowest value minus half of the group spacing, while values at the upper end were set to the highest value plus half of the group spacing.

Univariate analyses were performed using SPSS (IBM Corp. Released 2021; IBM SPSS Statistics for Windows, Version 28.0, Armonk, NY, USA). Binary logistic models with RCS terms were fitted using the “rms” package in R 4.0.2 [R Core Team (2023)]. R: A language and environment for statistical computing. R Foundation for Statistical Computing, Vienna, Austria].^2^ The “Predict” function in “rms” was used to present spline curves of predicted odds ratios of CAIT from RCS logistic models, and the likelihood ratio test (ANOVA function in “rms”) was used for overall (*p*_overall_) and non-linearity (*p*_non-linear_) testing. Statistical significance was assumed at a two-tailed *p-*value of less than 0.05.

### 2.4 Ethics statement

This study adhered to the principles of the Declaration of Helsinki and was approved by the Institutional Review Board (IRB) of Guilin Medical University. Because it was only a review of medical examination records from which the participants could not be identified, the IRB decided to waive the requirement to get informed consent.

## 3 Results

### 3.1 Baseline characteristics

A total of 214,092 routine physical examinations were exported from the hospital information system. After excluding individuals who did not meet the inclusion criteria, 111,494 examinations remained for the analysis (Supplementary Flow Chart). The characteristics of the participants according to the CAIT condition (yes or no) are shown in Supplementary Table S1. The median age of participants with CAIT was greater than that of those without it (*p *< 0.001). Participants with CAIT had higher BP measurements (SBP, DBP, PP, MAP) than their counterparts (all *p*-values < 0.001). Except for lymphocyte count, all other adjustment variables significantly differed between these two groups (Supplementary Table S1).

### 3.2 Non-linear associations between CAIT and BP measurements

**Figure 1:**
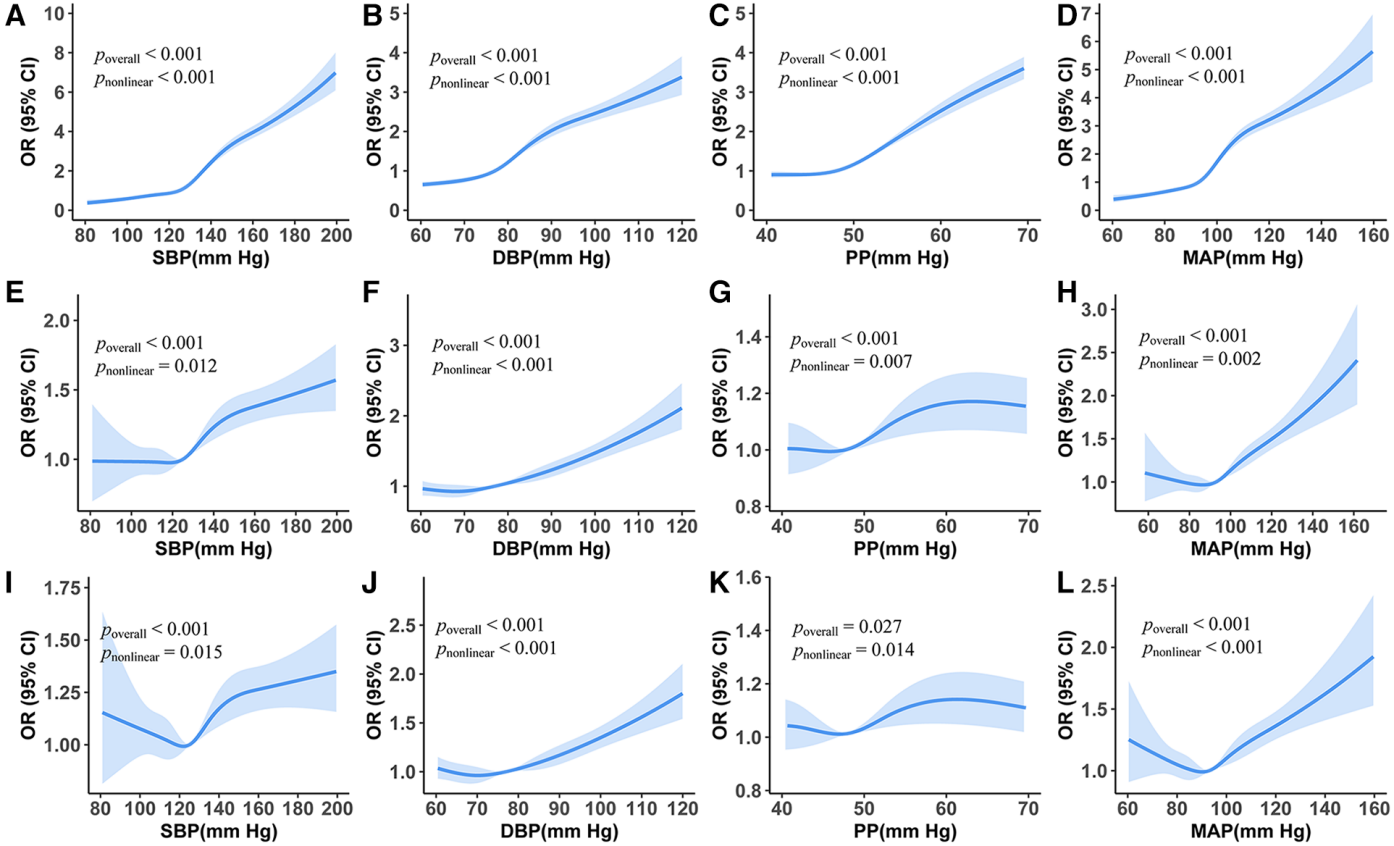
Association between carotid artery intima-media thickening and blood pressure measurements from restricted cubic splines logistic regression models. (**A–D**) Unadjusted odds ratios. (**E–H**) Adjusted odds ratios holding age and sex constant. (**I**–**L**) Odds ratio holds all the covariate variables constant.

When adding the RCS term of BP measurements in the multiple logistic regressions, all the *p*_non-linear_ values of BP measurements were less than 0.05. SBP had a J-curve association with the risk of CAIT: when SBP was lower than 120 mmHg, the risk decreased rarely; when SBP ranged between 120 and 140 mmHg, the risk increased rapidly; and it lowered growth speed when BP was above 160 mmHg. We also found a J-curve association between DBP and the risk of CAIT, and the risk was the lowest when DBP was around 70 mm Hg. PP also had a J-curve association with the risk of CAIT, reaching the highest risk at 65 mmHg. The J-curve associations between MAP and CAIT were also observed, with their turning point at around 90 mmHg (Figure 1).

From the RCS logistic regression model, we found that there were J-curve associations between CAIT and BP measurements even after adjusting for all selected adjustment variables (Figures 1I–L, all *p*_overall_ and *p*_non-linear_ values <0.05). The curve of SBP varied in shape and 95% CI as the number of adjustment variables increased. The lowest risk of CAIT was at an SBP level of approximately 120 mmHg (Figure 1I). Similarly, the lowest risk of CAIT was at a DBP level of approximately 72 mmHg (Figure 1J).

### 3.3 BMD of BP measurements and predicted risk of CAIT

**Figure 2:**
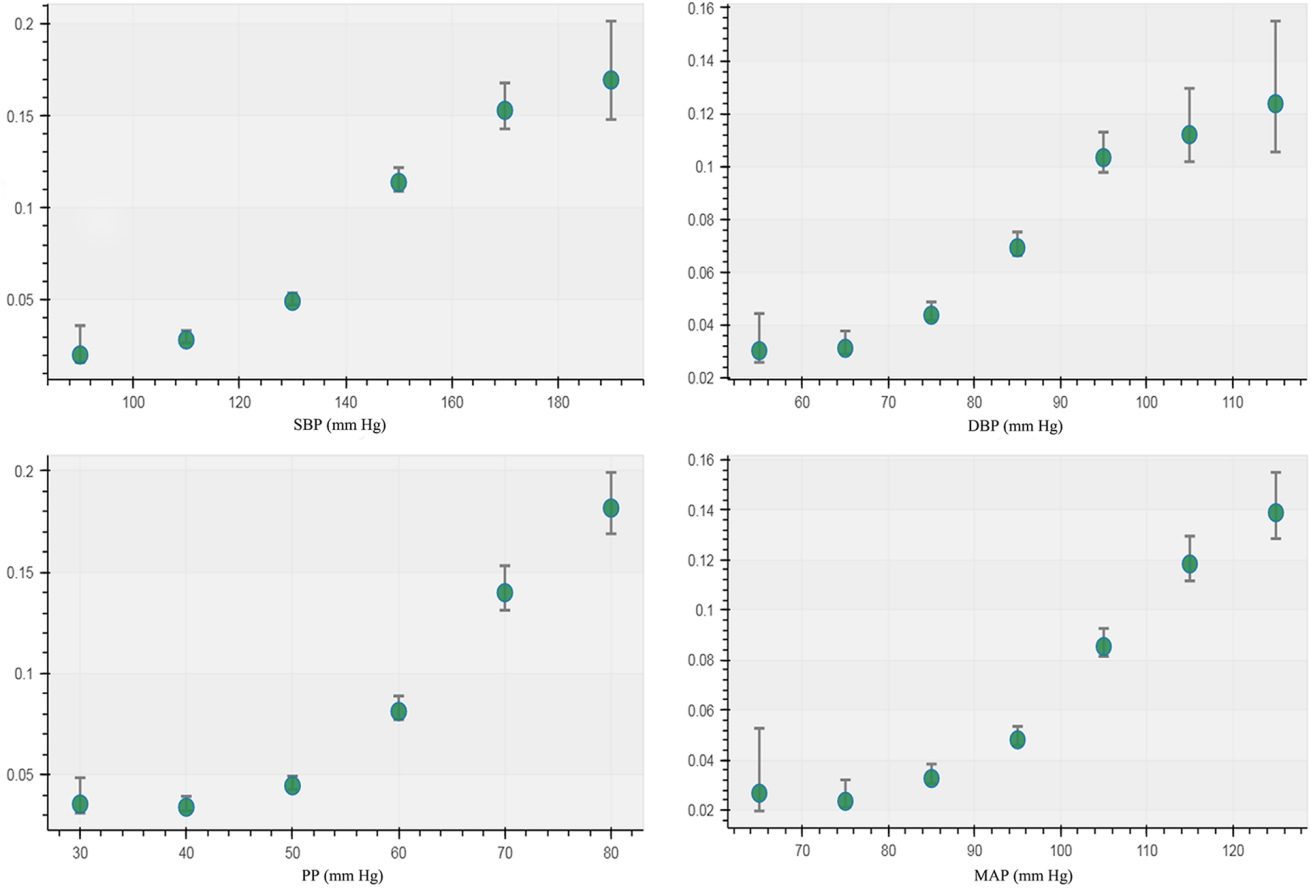
Incidence of carotid artery intima-media thickening in different groups of blood pressure measurements.

The incidence of CAIT in each group of BP measurements showed a J-curve association that was similar to the curve of the unadjusted RCS logistic model (Figure 2). The median (lower limit) risk BMR of 1% and 5% in SBP was 120.64 (118.58) and 138.88 (137.93) mmHg, respectively, while the median (lower limit) in DBP was 72.46 (70.08) mmHg and 87.58 (86.54) mmHg, respectively (Table 1). The predicted risk of CAIT at an SBP level of 100 mmHg was 2.52%, while the risk increased to 3.38% and 7.80% when SBP levels were 120 and 140 mmHg, respectively. Similarly, the predicted risk at DBP levels of 70, 80, and 90 mmHg was 3.58%, 5.48%, and 8.64%, respectively (Table 2).

**Table 1:** Benchmark dose of blood pressure at 1%, 5%, 10%, and 20% prevalence change of carotid artery intima-media thickening.

BMR	SBP (mmHg)		DBP (mmHg)		PP (mmHg)		MAP (mmHg)	
	BMD	BMDL	BMD	BMDL	BMD	BMDL	BMD	BMDL
1%	120.64	118.58	72.46	70.08	49.27	47.75	87.30	84.90
5%	138.88	137.93	87.58	86.54	60.33	59.66	102.36	101.45
10%	153.96	152.66	102.87	101.24	68.68	67.84	116.84	115.25
20%	184.43	182.27	128.62	125.26	83.59	82.38	135.86	133.99

**Table 2:** Bayesian benchmark dose model-predicted risks and odds ratios of carotid artery intima-media thickening at different blood pressure levels.

BP measurements	Value (mmHg)	Risk (%)	Lower (%)	Upper (%)	OR^a^
SBP	100	2.52	2.36	2.66	Ref
	120	3.38	3.27	3.50	1.35
	140	7.80	7.55	8.06	3.27
	160	14.00	13.00	14.00	6.30
	180	16.00	16.00	17.00	7.37
DBP	70	3.58	3.45	3.71	Ref
	80	5.48	5.31	5.55	1.56
	90	8.64	8.36	8.76	2.55
	100	11.00	11.00	11.00	3.33
	110	12.00	12.00	13.00	3.67
PP	40	3.45	3.31	3.59	Ref
	50	4.43	4.29	4.58	1.30
	60	8.11	7.81	8.41	2.47
	70	14.00	13.00	15.00	4.56
	80	18.00	17.00	19.00	6.14
MAP	70	2.45	2.23	2.65	Ref
	85	3.12	3.00	3.20	1.28
	100	6.50	6.31	6.71	2.77
	115	12.00	11.00	12.00	5.43
	130	14.00	14.00	15.00	6.48

^a^ Odds ratio was calculated by predicted risk.

## 4 Discussion

In this study, we demonstrated the non-linear association between BP measurements and the risk of CAIT onset and estimated the effect of the benchmark dose of BP on CAIT based on longitudinal data in a general population of southern China. First, the ordinary logistic model analysis (Supplementary Figure S1) showed that elevated SBP, DBP, and MAP were associated with an increased risk of CAIT with or without adjusting for confounding factors selected from three elastic regressions (Supplementary Table S1). However, after adjusting for all confounding variables, PP was not associated with CAIT. Second, the RCS logistic showed the J-curve association between SBP and DBP with CAIT even after adjusting for all confounding factors. Third, the BMDs of BP measurements at 1%, 5%, 10%, and 20% BMR were estimated, and the predicted risks of CAIT at different levels of BP were calculated.

Previous studies on the non-linear relationship between blood pressure and the risk of vascular disease have mostly focused on the cardio-cerebrovascular system. Some studies showed that cardiovascular disease and SBP have a J-curve association (16–18), but some clinical and observational studies have not found this relationship (30, 31). Because most of the patients undergoing cardio-cerebral angiography have suffered from myocardial infarction or stroke, needing angiography to determine whether the blood vessels form plaques or develop atherosclerosis, the relationships between BP measurements and macroangiosclerosis derived from data from these patients may not fully represent the general population because angiography is an invasive examination with complex procedures and is not performed in regular physical examinations. Instead, carotid ultrasonography for screening artery intima-media thickness in regular physical examinations is widely used; thus, the CAIT data would be more representative of the general population for predicting the future risk of cardiovascular disease and stroke based on BP. Several studies have shown that CAIT is associated with a higher risk of cardiovascular disease and stroke (32, 33), and the diagnosis of CAIT through B-mode ultrasound can effectively assess the risk of developing these diseases in advance (5). Therefore, exploring the relationship between CAIT and BP measurements may aid in controlling the adverse effects of blood pressure on blood vessels, especially in cardiovascular and cerebrovascular diseases. In this large population-based study, individuals with CAIT were included. We employed elastic net regression to select the appropriate adjustment variables (see Supplementary Materials) to fit RCS logistic regression and BBMD models to better explain the relationships between CAIT and BP measurements.

This study showed that the risk of CAIT increased with higher BP measurements in the ordinary logistic regression (see Supplementary Materials), which was consistent with previous observational reports (2, 34, 35). Although the J-curve splines of logistic models were observed in this study, the logistic model was essentially generalized linear. The J-curve splines of RCS logistic regressions in this study indicated that the turning points of BP should be estimated to prevent CAIT or other vessel damage. Elevated BP could increase both the pressure and tangential shear stress that distend the vessel wall, affecting smooth muscle cells and endothelial cells. Smooth muscle cells remodel and stiffen due to increased tone, resulting in thickening of the arterial wall (36); meanwhile, the endothelial cells sense shear stress and convert its stimulus into intracellular biochemical signals (37); these signals, along with cholesterol deposition in the arterial wall, promote chronic inflammation (38), finally resulting in plaque. This mechanism may partially explain the J-curve association between BP measurements and CAIT (39).

BMD was used as a departure point for deriving human health guidance values, such as reference doses in the chemical risk of the dose–response assessment. The Bayesian BMD method has the potential to incorporate prior information to make dose–response modeling more reliable and can provide distributional estimates for important quantities in dose–response assessment (29). In the present study, the median BMD (BMDL) of SBP at 1% BMR for added risk was 120.64 (118.58) mmHg, as analyzed by the BBMD models in the present study. This is very close to the normal SBP threshold recommended in the guidelines issued in 2017 (19) and is also close to the results of using the receiver operating characteristic curve of blood pressure to predict the CAIT boundary value (40), indicating that the rate of increase in CAIT occurrence risk changes at this departure point. According to the risk prediction of CAIT at different SBP levels, there was a 50% increase in the risk (3.38% vs. 2.25%) from 100 to 120 mmHg, but the risk increased by 131% (7.80% vs. 3.38%) from 120 to 140 mmHg. As the BMDL could be used to estimate a daily exposure level in the human population (including sensitive subgroups) that was likely to be associated with no appreciable risk of adverse effects over a lifetime (41), this result suggests that hypertensive individuals with more intensive SBP reduction to 120 mm Hg may experience a lower risk of CAIT and, furthermore, of macrovascular sclerosis. Previous studies have shown that there is a log-linear relationship between blood pressure and mortality from vascular disease, demonstrating that a 20 mmHg increase in usual SBP is associated with a twofold difference in risk (42). However, in this study, we found that the increment of risk above 140 mmHg is smaller than that below 140 mmHg. This difference may be caused by different outcome indicators.

The J-curve association between DBP and the risk of CAIT is consistent with the cardiovascular outcome (43). Although a previous study found no genetic evidence for a non-linear relationship between DBP and adverse cardiovascular disease (CVD) outcomes (44), another study pointed out that at different stages of atherosclerosis formation, the effect of too high or too low diastolic blood pressure was different (45), which indicated that the effect of DBP on CAIT was more complicated than that of SBP. This study revealed a DBP of 72.46 mmHg as the BMD for an added risk of 1% BMR, which is consistent with the result of a meta-analysis (42), suggesting the 70 mm Hg is the target of aggressive DBP decreasing level in hypertensive patients. However, as the coronary arteries are perfused predominantly during diastole, too low a DBP level could lead to hypoperfusion; therefore, 75–80 mmHg may be a better DBP target for hypertensive patients, even though at a DBP level of 80 mmHg, the predicted risk of CAIT was 5.48% (Table 2). Previous studies showed that intensive blood pressure control (<120/80 mmHg) could reduce the risk of cardiovascular events (46, 47). This is consistent with our study, but a randomized controlled trial showed that intensive blood pressure control should be avoided for acute ischemic stroke patients after endovascular thrombectomy (48). Our results suggest that this intensive target would reduce the risk of CAIT.

As age and sex may play a role in the non-linear association between BP and vessel damage (43), we further conducted an age- and sex-stratified analysis of the association between BP measurements and CAIT (Supplementary Figure S2) after observing the non-linear association in the age- and sex-adjusted model. The non-significant (e.g., in women aged 20–40 years) and non-linear (e.g., SBP in men aged 20–40 years) patterns in subgroups may suggest a varied association between BP measurements and the risk of CAIT, which needs further exploration in our future study.

Our research has some strengths. We employed RCS logistic regression to analyze longitudinal data, which not only strengthened the argument of cross-sectional data in previous studies but also addressed a limitation of ordinary logistic regression, which can only reflect the generalized linear relationship between the dependent variable and the independent variable. The departure points of BP measurements and CAIT estimated by the non-linear models of the BBMD weighted average are more reasonable than those from the single model in previous studies (40).

There were also some limitations in this study. First, as short-term blood pressure variability, which may increase the risk of CAIT (49), was not adjusted for, this may lead to a confounding effect on the associations between BP measurements and CAIT. Second, the data in this study did not include lifestyle information (e.g., smoking or drinking status), which may also affect the association between BP measurements and CAIT. Third, bias may exist in BMD analysis as we did not adjust for the selected covariates due to the limitations of the online tool. Finally, we did not explore the interactions between BP measurements and other adjustment variables.

In conclusion, the J-curve associations of SBP and DBP with the risk of CAIT were observed in the general population in southern China, and the optimal blood pressure control target for significantly reducing the risk of CAIT was determined as to be 120/75–80 mmHg.

## Data Availability

The datasets presented in this article are not readily available because they are for internal use only and are not suitable for sharing. Requests to access the datasets should be directed to Shengkui Tan, q527150899@outlook.com.

## Footnotes

^1^Available online at: https://benchmarkdose.com.

^2^Available online at: https://www.r-project.org/.
