# Supplementary material for: Non-linear association and benchmark dose of blood pressure on carotid artery intima-media thickening in a general population of southern China
Source: Front Cardiovasc Med. 2024 May 13;11:1325947. doi: 10.3389/fcvm.2024.1325947 (PMC11128656; doi:10.3389/fcvm.2024.1325947)
Supplement: Supplementary file 1 [file Datasheet1.docx]

**Supplementary materials**

**Nonlinear Association and Benchmark Dose of Blood Pressure on Carotid Artery Intima-media Thickening in a General Population of Southern China**

Linyuan Qin^a,b^, Xiaoyan Wu^a,b^, Chao Tan^a,b^, Zhengbao Zhang^a,b^, You Li^a,b^, Xiaonian Zhu^a,b^, Shenghua Qin^c^, Shengkui Tan^a,b,d*^

^a^ Department of epidemiology and health statistics, school of public health, Guilin medical university, Zhiyuan Road, Guilin City, Guangxi Province, China, 541000

^b^ Guangxi key laboratory of Environmental Exposomics and Entire Lifecycle Health, Zhiyuan Road, Guilin City, Guangxi Province, China, 541000, China

^c^ Guilin Peoples’ Hospital, Wenming Road, Guilin City, Guangxi Province, China, 541000, China

^d^ Youjiang Medical University For Nationalities, Baise, Chengxiang Road, Baise City, Guangxi Province, China, 533000

*Corresponding author: Shengkui Tan, Email: [Q527150899@outlook.com](mailto:Q527150899@outlook.com), Add.: Zhiyuan Road, Guilin City, Guangxi Province, China, 541000

## This work was supported by Construction and application of chronic disease risk model based on physical examination cohort in Guilin, Guilin Innovation Platform and Talent Plan in 2022, [20220120-2].

## 1. Statistical analysis

Comparisons of continuous variables between groups were done using t–tests or Mann-Whitney U tests according to their skewness. We used SBP, DBP, PP, and MAP as the main explanatory variables, and used the carotid artery intima-media thickening (CAIT) condition (yes vs. no) as the dependent variable to fit the models. To avoid collinearity and redundancies for explanatory variables and covariate variables, we fitted the models separately for every BP measurement and employed generalized linear models with the elastic net penalty to select the most important covariate variables for multivariate models. Three elastic net logistic models were used: LASSO, ridge, and the elastic net model with mixing parameter α set to 0.5. To include adequate adjustment variables in models, we used one standard error of the minimum mean cross-validated (“lambda.1se”) in elastic net regressions. The ordinary unadjusted and adjusted binary logistic regression models were employed to access the associations of BP measurements and CAIT. The results of logistic models were presented as spline curves based on main effects with 95% confidence intervals (CIs). elastic net logistic regressions were fitted using the package “glmnet” in R 4.0.2 (R Core Team (2023). R: A language and environment for statistical computing. R Foundation for Statistical Computing, Vienna, Austria.)^[[1]](#footnote-1)^. Statistical significance was assumed at a two-sided *p-*value of less than 0.05.

## 2. Results

## 2.1 Flow chart of this study

A total of 214092 regular physical examinations were exported from the hospital information system in the Affiliated Hospital of Guilin Medical University during 2011-2016.

A total of 211103 examinations left after excluding individuals <18 years.

A total of 183327 examinations left after excluding individuals that not performed carotid artery ultrasound.

A total of 168752 examinations left after excluding individuals who participated only once in the physical examinations.

A total of 111494 examinations left for the analysis after excluding individuals who have CAIT in the first examination.

## 2.2 Baseline characteristics and covariate variables selected

The characteristics of the participants according to CAIT condition (yes or no) are shown in Table 1. The median age of participants with CAIT was larger than that of those without it (*p* < 0.001). Participants with CAIT had higher BP measurements (SBP, DBP, PP, MAP) than their opposites (all *p*-values < 0.001). Except for lymphocyte count, all other adjustments significantly differed between these two groups. Combined with all three results of elastic net regressions, we finally determined that age, gender, BMI, fasting blood glucose, low-density lipoprotein cholesterol, serum uric acid, albumin, alkaline phosphatase, homocysteine, and fatty liver as the adjustment variables for further analysis, as their coefficients did not shrink to zero as lambda increased (Table 1).

Table 1 Characteristics of participants without (no) or with (yes) carotid artery intima-media thickening and coefficients of three elastic net regressions ^a^

| Characteristics | Total | No (105286 examines) | Yes (6208 examines) | Z/χ^2^ | *P* | *Lasso^b^* | *elastic^b^* | *ridge^b^* | *selected^c^* |
| --- | --- | --- | --- | --- | --- | --- | --- | --- | --- |
| Age(years) | 43.0(33.0~53.0) | 42.0(33.0~52.0) | 60.0(52.0~68.0) | 86.933 | <0.001 | 0.0727 | 0.0638 | 0.0630 | yes |
| Pulse(beats/min) | 79.0(72.0~86.0) | 79.0(72.0~86.0) | 78.0(71.0~86.0) | 2.607 | 0.009 |  |  |  |  |
| SP(mmHg) | 125.0(114.0~136.0) | 124.0(113.0~135.0) | 136.0(124.0~151.2) | 48.024 | <0.001 |  | 0.0018 | 0.0020 | yes |
| DP(mmHg) | 77.0(69.0~84.0) | 77.0(69.0~84.0) | 82.0(74.0~90.0) | 34.112 | <0.001 | 0.0004 |  |  | yes |
| MAP (mmHg) | 93.0(84.3~101.3) | 92.7(84.0~100.7) | 100.3(91.3~110.0) | 43.229 | <0.001 | 0.0046 | 0.0032 | 0.0028 | yes |
| PP (mmHg) | 48.0(42.0~54.0) | 47.5(42.0~54.0) | 53.0(45.0~64.0) | 39.460 | <0.001 |  |  |  | yes |
| BMI(kg/m^2^) | 24.0(21.7~26.0) | 23.9(21.7~25.9) | 24.7(22.9~26.8) | 24.157 | <0.001 |  |  |  | yes |
| WBC (10^9^/L) | 6.6(5.6~7.6) | 6.6(5.6~7.6) | 6.8(5.8~7.9) | 11.514 | <0.001 |  |  |  |  |
| RBC (10^12^/L) | 4.8(4.5~5.2) | 4.8(4.5~5.2) | 4.8(4.5~5.1) | 2.866 | 0.004 |  |  |  |  |
| Hemoglobin (g/L) | 142.0(132.0~153.0) | 142.0(132.0~153.0) | 145.0(135.0~155.0) | 12.995 | <0.001 |  |  |  |  |
| Platelet (10^9^/L) | 225.0(193.0~259.0) | 226.0(193.0~259.0) | 217.0(185.0~252.0) | 12.355 | <0.001 |  |  |  |  |
| Platelet distribution width (%) | 11.2(10.3~12.2) | 11.2(10.3~12.2) | 11.1(10.1~12.1) | 6.331 | <0.001 |  |  |  |  |
| Lymphocyte (10^9^/L) | 2.2(1.9~2.6) | 2.2(1.9~2.6) | 2.2(1.9~2.7) | 0.874 | 0.382 |  |  |  |  |
| Monocyte (10^9^/L) | 0.4(0.3~0.5) | 0.4(0.3~0.5) | 0.4(0.3~0.5) | 14.298 | <0.001 |  |  |  |  |
| Neutrophil (10^9^/L) | 3.6(3.0~4.4) | 3.6(3.0~4.4) | 3.8(3.1~4.6) | 11.027 | <0.001 |  |  |  |  |
| Average RBC distribution width CV (%) | 12.9(12.4~13.5) | 12.9(12.4~13.5) | 13.1(12.6~13.7) | 19.268 | <0.001 |  |  |  |  |
| Fasting blood glucose (mmol/L) | 5.4(5.1~5.8) | 5.4(5.1~5.8) | 5.7(5.3~6.4) | 42.971 | <0.001 | 0.0562 | 0.0627 | 0.0610 | yes |
| Cholesterol (mmol/L) | 4.9(4.3~5.4) | 4.8(4.3~5.4) | 5.1(4.5~5.7) | 20.029 | <0.001 |  |  |  |  |
| Triglyceride (mmol/L) | 1.2(0.8~1.7) | 1.2(0.8~1.7) | 1.4(1.0~2.0) | 21.442 | <0.001 |  |  |  |  |
| High density lipoprotein cholesterol (mmol/L) | 1.4(1.1~1.6) | 1.4(1.1~1.6) | 1.3(1.1~1.5) | 17.402 | <0.001 |  |  |  |  |
| Low density lipoprotein cholesterol (mmol/L) | 3.2(2.6~3.7) | 3.2(2.6~3.7) | 3.4(2.9~4.1) | 25.241 | <0.001 | 0.0502 | 0.0431 | 0.0341 | yes |
| Blood urea nitrogen (mmol/L) | 4.5(3.8~5.3) | 4.5(3.8~5.2) | 5.0(4.2~6.0) | 34.485 | <0.001 |  |  |  |  |
| Serum creatinine  (μmol/L) | 72.0(60.0~84.5) | 71.4(60.0~84.0) | 78.4(67.0~91.0) | 30.345 | <0.001 |  |  |  |  |
| Serum uric acid  (μmol/L) | 328.7(270.0~393.0) | 326.2(268.3~391.0) | 363.9(305.0~427.1) | 30.198 | <0.001 | 0.0007 | 0.0007 | 0.0007 | yes |
| Alanine aminotransferase (U/L) | 16.6(11.6~24.5) | 16.5(11.5~24.5) | 17.8(13.2~24.9) | 10.763 | <0.001 |  |  |  |  |
| Aspartate aminotransferase (U/L) | 19.3(16.3~23.2) | 19.3(16.3~23.1) | 20.3(17.4~24.3) | 15.980 | <0.001 |  |  |  |  |
| Total bilirubin  (μmol/L) | 10.8(8.2~13.1) | 10.8(8.2~13.1) | 10.7(8.4~13.5) | 4.191 | <0.001 |  |  |  |  |
| Direct bilirubin  (μmol/L) | 3.8(3.0~4.4) | 3.8(3.0~4.4) | 3.8(3.1~4.6) | 5.401 | <0.001 |  |  |  |  |
| Indirect bilirubin  (μmol/L) | 7.2(5.3~8.8) | 7.2(5.2~8.8) | 7.0(5.3~9.0) | 2.761 | 0.006 |  |  |  |  |
| Total protein (g/L) | 75.4(73.4~77.6) | 75.4(73.4~77.6) | 75.2(72.8~77.5) | 8.089 | <0.001 |  |  |  |  |
| Albumin (g/L) | 46.2(44.7~47.8) | 46.2(44.8~47.9) | 45.3(43.6~47.1) | 28.841 | <0.001 | -0.0063 | -0.0132 | -0.0104 | yes |
| Globulin (g/L) | 29.2(27.4~31.3) | 29.2(27.4~31.3) | 30.1(27.7~31.9) | 15.372 | <0.001 |  |  |  |  |
| γ-Glutamyl transpeptidase (U/L) | 36.8(23.8~36.8) | 36.8(23.8~36.8) | 36.0(23.8~40.0) | 2.130 | 0.033 |  |  |  |  |
| Alkaline phosphatase (U/L) | 71.4(66.5~71.4) | 71.4(66.5~71.4) | 71.4(66.0~84.0) | 20.927 | <0.001 | 0.0017 | 0.0014 | 0.0011 | yes |
| Homocysteine (μmol/L) | 12.3(12.3~12.3) | 12.3(12.3~12.3) | 12.3(11.5~15.0) | 22.277 | <0.001 | 0.0047 | 0.0087 | 0.0082 | yes |
| Gender: Female | 55484 | 55484(96.4) | 2070(3.6) | 879.310 | <0.001 | 0.3631 | 0.3073 | 0.2897 | yes |
| Male | 49802 | 49802(92.3) | 4138(7.7) |  |  |  |  |  |  |
| Fatty liver: No | 86144 | 86144(95.1) | 4462(4.9) | 380.756 | <0.001 | 0.0253 | 0.0004 |  | yes |
| Yes | 19142 | 19142(91.6) | 1746(8.4) |  |  |  |  |  |  |
| Bile duct stones: No | 103161 | 103161(94.4) | 6115(5.6) | 8.138 | 0.004 |  |  |  |  |
| Yes | 2125 | 2125(95.8) | 93(4.2) |  |  |  |  |  |  |
| Gallstone: No | 96604 | 96604(94.8) | 5317(5.2) | 278.499 | <0.001 |  |  |  |  |
| Yes | 8682 | 8682(90.7) | 891(9.3) |  |  |  |  |  |  |
| Gallbladder polyps:  No | 103967 | 103967(94.4) | 6137(5.6) | 0.567 | 0.452 |  |  |  |  |
| Yes | 1319 | 1319(94.9) | 71(5.1) |  |  |  |  |  |  |
| Renal calculi: No | 97595 | 97595(94.8) | 5380(5.2) | 302.333 | <0.001 |  |  |  |  |
| Yes | 7691 | 7691(90.3) | 828(9.7) |  |  |  |  |  |  |
| Renal cyst: No | 100515 | 100515(94.9) | 5358(5.1) | 1027.584 | <0.001 |  | 0.0049 |  |  |
| Yes | 4771 | 4771(84.9) | 850(15.1) |  |  |  |  |  |  |
| Hepatic cyst: No | 98875 | 98875(94.9) | 5359(5.1) | 554.296 | <0.001 |  |  |  |  |
| Yes | 6411 | 6411(88.3) | 849(11.7) |  |  |  |  |  |  |

^a^ Median (P25~P75) for continuous variables and n (%) for categorical variables.

^b^ Coefficients of LASSO, elastic net and ridge regression models. Blank indicates the coefficient shank to zero.

^c^ All BP measurements were selected in future analysis as they were our main interested variables. Renal cyst was not selected as there was neither epidemic evidence nor pathological explanation supporting its effect on carotid artery intima-media thickening.

## 2.3 Associations of CAIT and BP measurements

Ordinary binary logistic regression showed that all BP measurements except PP were positively associated with the risk of CAIT before and after adjusting for all the adjustment variables. (all p-values < 0.001, Figure 1). As the number of adjustment variables in the model increased, the linear curve became more visible. The curves become roughly straight after adjusting for gender and age. After controlling for all of the specified adjustment variables, the linear trends of SBP, DBP, and MAP became more apparent, however, PP was not significantly linked with CAIT. (Figure 1).


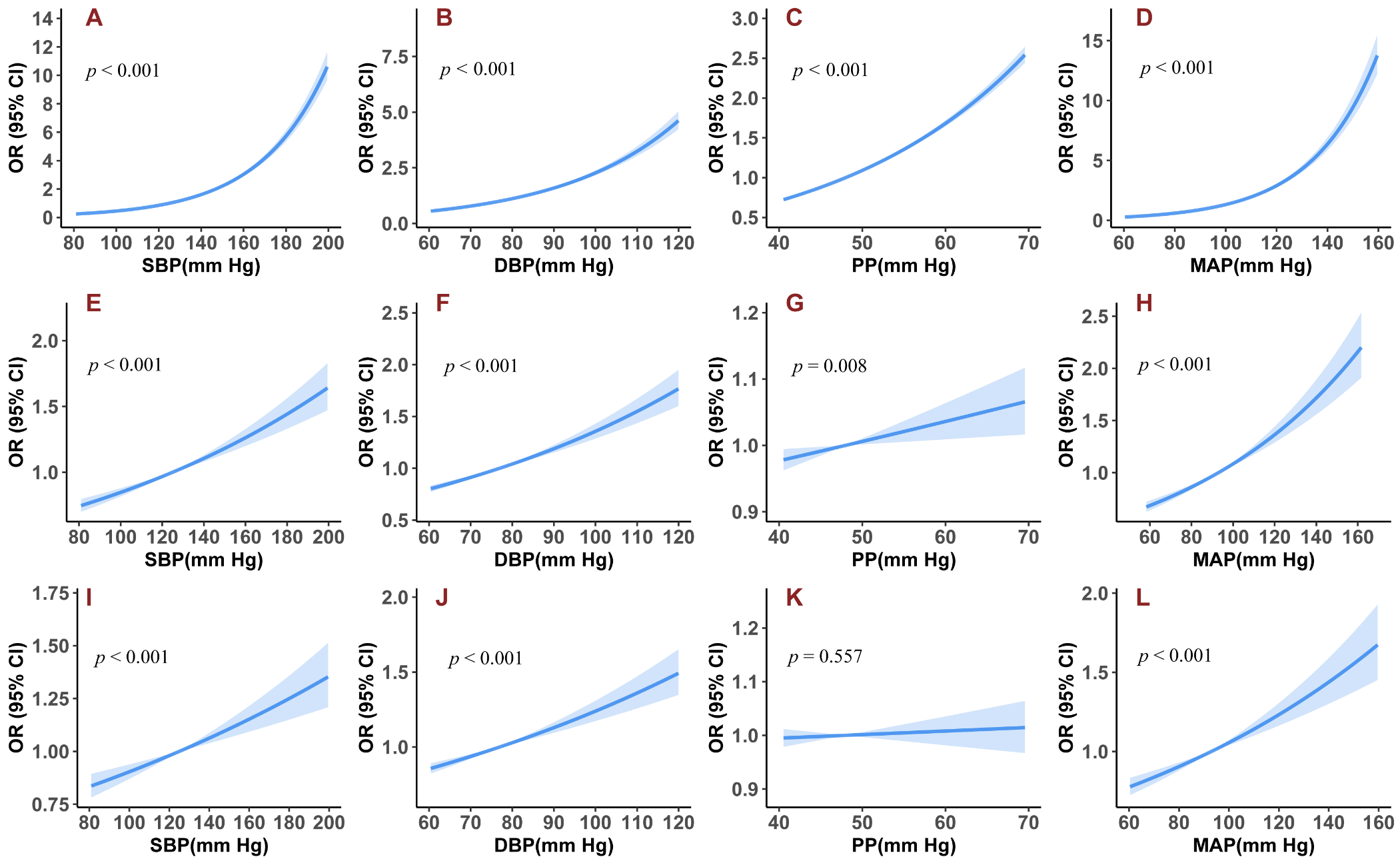


Figure 1 Association of carotid artery intima-media thickening and blood pressure measurements from ordinary logistic regression models.

A, B, C and D show the unadjusted odds ratios; E, F, G and H show the adjusted odds ratios holding age and sex constant; and I, J, K and L show the adjusted odds ratios of hold all the covariate variables constant.


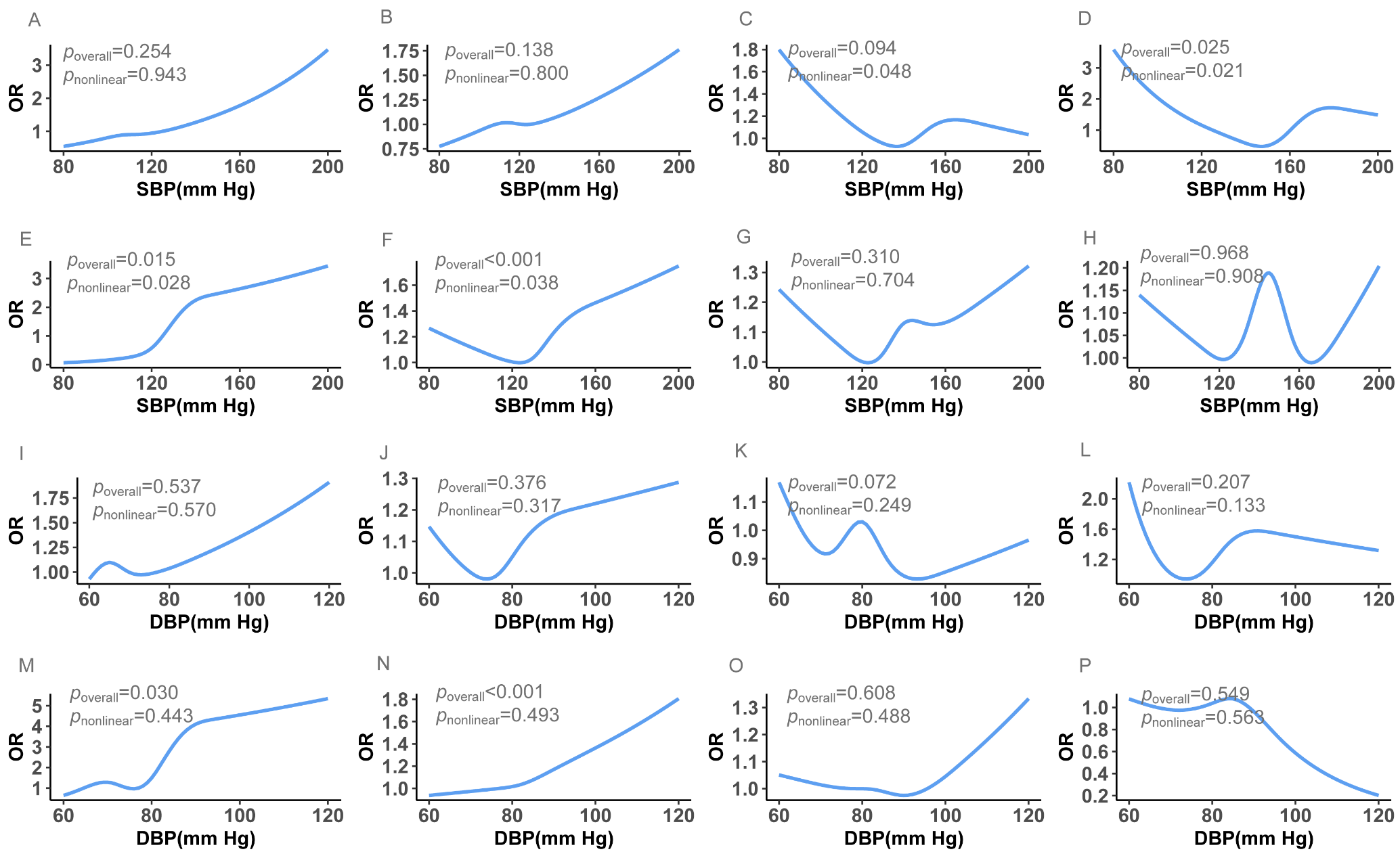


Figure 2 Age-sex specific association of carotid artery intima-media thickening and blood pressure measurements from restricted cubic splines logistic regression models holding all the covariate variables constant .

A, B, C and D represent female aged <20, 20-40, 41-60 and >60 respectively; E, F, G and H represent male aged <20, 20-40, 41-60 and >60 respectively; I, J, K and L represent female aged <20, 20-40, 41-60 and >60 respectively; M, N, O and P represent male aged <20, 20-40, 41-60 and >60 respectively.

1. Website URL: <https://www.r-project.org/> [↑](#footnote-ref-1)
